# Synergy Effect of High-Stability of VS_4_ Nanorods for Sodium Ion Battery

**DOI:** 10.3390/molecules27196303

**Published:** 2022-09-24

**Authors:** Yi Chen, Haimei Qi, Jie Sun, Zhibin Lei, Zong-Huai Liu, Peng Hu, Xuexia He

**Affiliations:** 1 Key Laboratory of Applied Surface and Colloid Chemistry, Ministry of Education, Shaanxi Normal University, Xi’an 710062, China; 2 Shaanxi Engineering Lab for Advanced Energy Technology, School of Materials Science and Engineering, Shaanxi Normal University, Xi’an 710119, China; 3 School of Physics, Northwest University, Xi’an 710069, China

**Keywords:** Sodium-ion batteries, two-dimensional material, vanadium tetrasulfide, synergy effect, morphology control

## Abstract

Sodium-ion batteries (SIBs) have attracted increasing interest as promising candidates for large-scale energy storage due to their low cost, natural abundance and similar chemical intercalation mechanism with lithium-ion batteries. However, achieving superior rate capability and long-life for SIBs remains a major challenge owing to the limitation of favorable anode materials selection. Herein, an elegant one-step solvothermal method was used to synthesize VS_4_ nanorods and VS_4_ nanorods/reduced graphene oxide (RGO) nanocomposites. The effects of ethylene carbonate/diethyl carbonate(EC/DEC), ethylene carbonate/dimethyl carbonate(EC/DMC), and tetraethylene glycol dimethyl ether (TEGDME) electrolytes on the electrochemical properties of VS_4_ nanorods were investigated. The VS_4_ nanorods electrodes exhibit high specific capacity in EC/DMC electrolytes. A theoretical calculation confirms the advance of EC/DMC electrolytes for VS_4_ nanorods. Significantly, the discharge capacity of VS_4_/RGO nanocomposites remains 100 mAh/g after 2000 cycles at a large current density of 2 A/g, indicating their excellent cycling stability. The nanocomposites can improve the electronic conductivity and reduce the Na^+^ diffusion energy barrier, thereby effectively improving the sodium storage performance of the hybrid material. This work offers great potential for exploring promising anode materials for electrochemical applications.

## 1. Introduction

Since the successful preparation of graphene in 2004 [1,2], two dimensional transition metal dichalcogenides (TMDs) have aroused increasing research interests for their potential applications in the field of electrochemical storage owing to the unique layered structure and outstanding physical and chemical properties [3,4,5]. Currently, lithium-ion batteries (LIBs) dominate portable electronic devices and smart grids ascribed to their high energy density and excellent long cycling stability [6,7,8]. However, the high cost of lithium caused by limited resource and uneven distribution of lithium hinders the further development in large-scale storage systems. Therefore, it is of great importance to develop desirable battery storage technologies with low prices as an alternative to LIBs [9,10,11]. Sodium-ion batteries (SIBs) have attracted rapidly growing attention for their earth abundance and similar chemistry intercalation mechanism compared with LIBs, and are looking forward to be one of the most promising next generation energy storage devices [12,13]. Despite its great potential, sluggish reaction kinetics caused by the large ionic radius and the lack of suitable electrode materials limit the practical application of SIBs [14,15]. Hence, it is of great importance to explore favorable SIBs anode materials with high specific capacity, excellent rate capability and superior long life cycles.

Among the studied TMDs anode materials, vanadium-based chalcogenides [16,17] have received widespread interest due to their unique layered crystal structure and high electronic conductivity [18,19,20]. Specifically, vanadium sulfide (VS_4_) with a monoclinic phase possesses a unique linear-chain structure consisting of V^4+^ and sulfur dimers (S_2_^2−^) extending along the c-axis direction, and the individual atomic chains are bonded by weak interchain van der Waals forces, forming a loosely stacked framework [21]. The large open channels (interchain distance of VS_4_ is 0.583 nm) can provide abundant active sites for Na-ion diffusion and storage [22]. In addition, VS_4_ has a high theoretical capacity of 1196 mAh/g and is considered as a promising candidate anode material for SIBs [21,23]. Nevertheless, the disadvantages of VS_4_ electrode materials including low conductivity and large volume expansion lead to poor rate performance and cycling stability during the discharge/charge process, hindering the practical energy storage applications. To promote the electrochemical performance, it has been widely accepted that one strategy is to construct nanosized electrode materials due to the sufficient redox sites and to shorten the electron/ion diffusion distance during sodiaion/desodiation [24,25]. The second strategy is to incorporate nanomaterials into highly conductive graphene that can effectively enhance the conductivity and maintain the whole structure stability of electrodes [26,27,28,29].

In this work, we report the fabrication of uniform VS_4_ nanorods by a facile solvothermal method. After introducing graphene oxide into the reaction system, VS_4_ nanorods/reduced graphene oxide hybrids (denoted as VS_4_/RGO) were successfully obtained after subsequent annealing treatment. The VS_4_ nanorods have an average diameter of 30 nm, and the small VS_4_ nanorods grown on the conductive support of RGO nanosheets can elevate the electronic conductivity and effectively interrupt their aggregation. Furthermore, three typical electrolytes [30] are optimized to achieve the high Na ions storage performance for VS_4_ nanorods. Using ethylene carbonate/dimethyl carbonate (EC/DMC) as the electrolyte, the as-prepared novel VS_4_/RGO nanocomposites display superior cycling stability with the discharge specific capacity of 100 mAh/g after 2000 cycles at a current density of 2 A/g. The improved performance is attributed not only to the conductive network of large-size RGO nanosheets, but also to the EC/DMC electrolyte with high ionic conductivity, low solvation energy and Lowest Unoccupied Molecular Orbital (LUMO) energy.

## 2. Results

**Figure 1:**
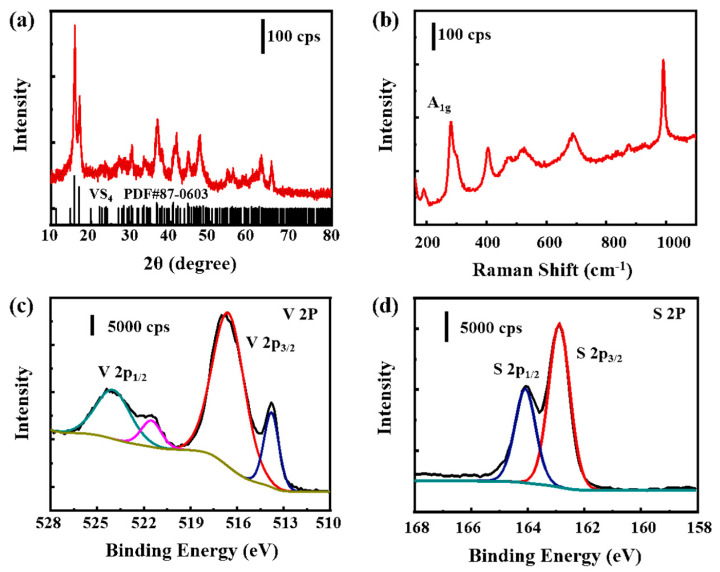
(**a**) XRD pattern, (**b**) Raman spectrum and (**c**), (**d**) XPS spectra of VS_4_ nanorods.

The XRD diffraction pattern of VS_4_ nanorods is shown in Figure 1a. All the diffraction peaks are perfectly matched to the monoclinic phase of VS_4_ (JCPDS no. 87-0603, space group I2/c, lattice constants a = 0.677 nm, b = 1.042 nm, c = 1.211 nm), indicating the high phase purity [21,23]. The two strongest characteristic diffraction peaks at 2θ = 15.7° and 17° are in line with the (110) and (020) crystal planes. A Raman spectrum of VS_4_ nanorods shows that two typical bands at 190 and 279 cm^−1^ are attributed to the stretching and bending vibration modes of the V−S bonds, respectively (Figure 1b) [31].

The XRD and Raman spectroscopy analyses confirm that the prepared sample is monoclinic VS_4_. The V 2_p_ X-ray photoelectron spectroscopy (XPS) of the material in Figure 1c shows two peaks at 517.3 and 524.3 eV, which are ascribed to the 2p_3/2_ and 2p_1/2_ components of V^4+^, respectively. Two other peaks at 513.9 and 521.5 eV correspond to the V−C bond, which indicates that the VS_4_ nanorods are anchored on the graphene surface through it. The scanning at S 2p region demonstrates the existence of S_2_^2−^ dimers (Figure 1d), and the peaks at 162.9 and 164.0 eV are ascribed to S 2p_3/2_ and S 2p_1/2_ bands, respectively.

**Figure 2:**
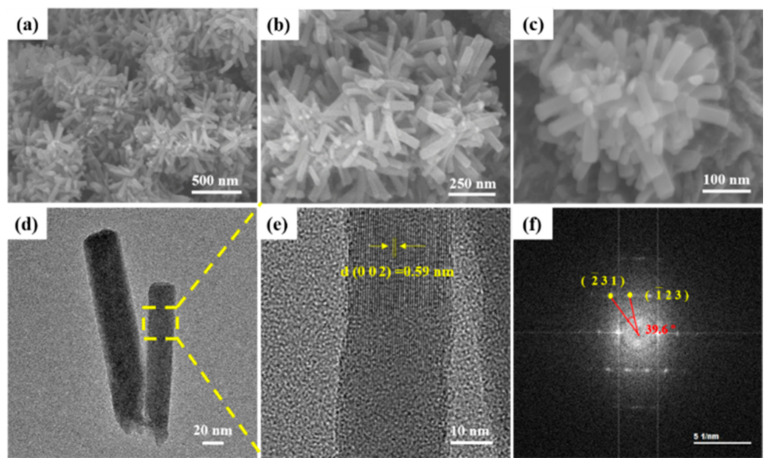
The morphology features and microstructures of VS_4_ nanorods: (**a**–**c**) FESEM images with different magnifications; (**d**) TEM image; (**e**) HRTEM image of lattice spacing; (**f**) the fast-Fourier transform (FFT).

As shown in Figure 2, the morphology and microstructure of the as-prepared VS_4_ nanorods were performed by field emission scanning electron microscopy (FESEM) and transmission electron microscopy (TEM). The obtained samples are composed of surface-smoothly and well-dispersed short nanorods with an average diameter about 30 nm. Moreover, these nanorods are randomly stacked together to form large clusters, which may be caused by the anisotropy of the monoclinic phase VS_4_ (Figure 2a–c). The TEM image of the products exhibits a rod-like structure. A lattice space of about 0.59 nm is observed by high-resolution transmission electron microscopy (HRTEM), which is consistent with the (002) plane of VS_4_. The fast-Fourier transform (FFT) (Figure 2f) further confirms the monoclinic phase of VS_4_.

**Figure 3:**
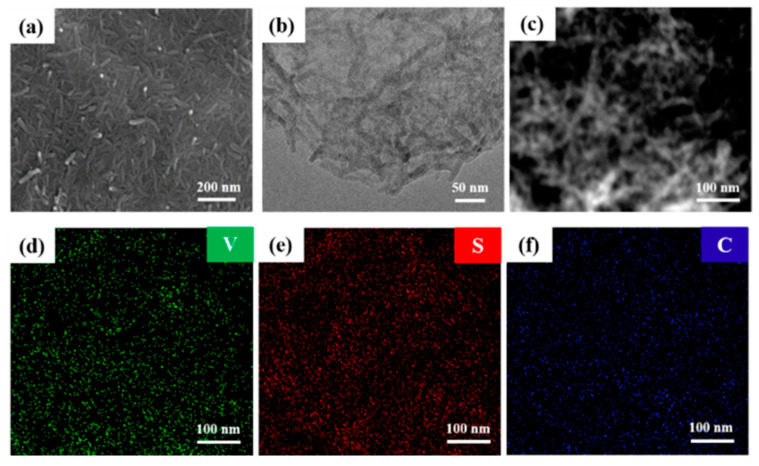
The morphology features of VS_4_/RGO nanocomposites: (**a**) FESEM images; (**b**) TEM images; (**c**–**f**) EDX elemental mapping images of VS_4_/RGO nanocomposites:(**d**) V, (**e**) S, (**f**) C.

The successful formation of the VS_4_/RGO nanocomposite is verified by Raman spectrum analysis in the range of 180–1800 cm^−1^ (Figure S1, Supplementary Materials). The two typical bands at 1340 cm^−1^ and 1585 cm^−1^ are designated as the D band and G band of RGO, respectively. The D band is associated with edge defects and disordered carbon in hexagonal graphene layers, while the G band is attributed to the in-plane stretching vibration of the sp^2^ carbon atoms hybrid and highly ordered graphite [23,32,33]. The two bands at 190 and 279 cm^−1^ in the low wavenumber region are the vibration dispersion characteristics of VS_4_. Figure 3a shows the FESEM images of VS_4_/RGO nanocomposites. Obviously, the surface of RGO with the large wrinkled sheet structure is not smooth in the hybrid materials. The substantial VS_4_ nanorods are grown on the large RGO nanosheets with uniform distribution, indicating that RGO sheets with rich oxygen-containing functional groups not only support the nucleation and growth of VS_4_, but also effectively alleviate the agglomeration of VS_4_ nanorods in the VS_4_/RGO nanocomposite. The TEM characterization of VS_4_/RGO nanocomposites are shown in Figure 3b. The VS_4_ nanorods are randomly and uniformly dispersed on the surface of the large-sized RGO nanosheets with typical wrinkled structure morphology, which is closely related to the anisotropy of the monoclinic phase VS_4_. The introduction of RGO as a large-area conductive channel plays an important role in enhancing the electrical conductivity and improving the structural stability of VS_4_. The energy dispersive X-ray (EDX) elemental mappings analysis reveals that these elements of V, C and S are uniformly distributed throughout the entire VS_4_/RGO nanocomposite (Figure 3c–f). The growth process of the prepared VS_4_/RGO nanocomposite should be that oxygen-containing functional groups provided a large number of growth sites for VS_4_, so that the VS_4_ nanorods grew uniformly on the RGO sheet, thereby obtaining uniform composites of RGO and VS_4_. During the hydrothermal reaction, VS_4_ gradually nucleated and grew on the surface of GO, while GO lost most of the oxygen-containing functional groups and was reduced to graphene (RGO).

**Figure 4:**
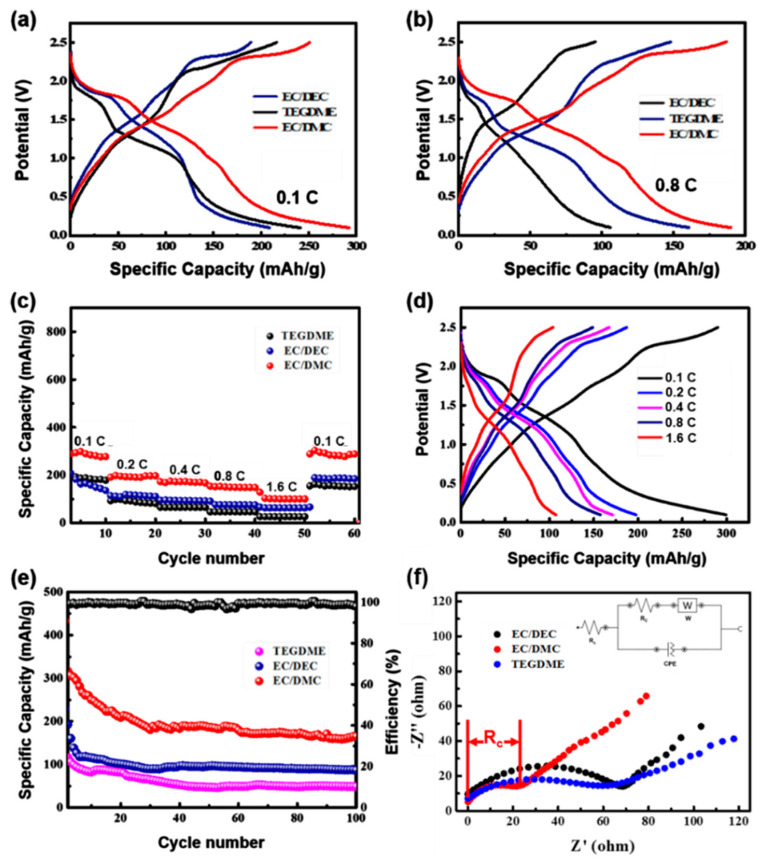
Electrochemical measurements of VS_4_ nanorods with different electrolytes: (**a**,**b**) the charge-discharge curves at the current density of 0.1 C and 0.8 C, respectively; (**c**) rate performance; (**d**) the charge-discharge curves at different current densities of VS_4_ nanorods in the EC/DMC electrolyte; (**e**) the cycling test at 0.4 C for 100 cycles; (**f**) EIS Nyquist impedance spectra.

Electrolytes are essential for the proper functioning of any battery technology, and the emerging Na-ion technology is no exception. Hence, a major focus on battery research is to identify the most appropriate formulation so as to minimize interface reactions and enhance both cell performances and safety aspects. While a number of efforts are being directed to the search for new electrode materials for sodium ion batteries, studies dealing with the electrolyte are much scarcer. Still, some available reports demonstrate that the solid electrolyte interphase (SEI) formed on carbonaceous electrodes is markedly different for sodium- and lithium-based electrolytes, even those using the same solvent. To investigate the effect of different electrolytes on the Na-ion storage performance of VS_4_ nanorods electrodes, CR2032 coin-type cells were assembled to obtain the superior electrochemical properties by using 1 M NaClO_4_ in ethylene carbonate/diethyl carbonate (1:1, *v*:*v*; EC/DEC), ethylene carbonate/dimethyl carbonate (1:1, *v*:*v*; EC/DMC), and tetraethylene glycol dimethyl ether (TEGDME) as electrolytes. The galvanostatic charge/discharge (GCD) profiles of pure VS_4_ nanorods with the three selected electrolytes in the voltage range of 0.1–2.5 V at the current density of 0.1 C and 0.8 C are shown in Figure 4a–b. When the EC/DMC based electrolyte is used, the discharge specific capacity of VS_4_ nanorods is 288 mAh/g at 0.1 C, which is higher than the other two electrolytes (Figure 4a). From Figure 4b and Table S1, taking the charge-discharge curve with a current density of 0.8 C as an example, when the electrolyte is EC/DMC, the Coulombic efficiency of the material is the highest (98%), indicating that the electrolyte can obtain the best electrochemical performance, and its charge-discharge capacity is 186, 190 mAh/g, respectively; when the electrolyte is EC/DEC, its coulombic efficiency is 92%, and the charge-discharge capacity is 148, 161 mAh/g, respectively; when the electrolyte is TEGDME, the coulombic efficiency is the lowest (90%), and the charge-discharge capacity is 96, 106 mAh/g. 

The rate performance of three electrolytes is summarized and compared at various current densities from 0.8 C to 1.6 C in Figure 4c. The capacities of VS_4_ nanorods in the EC/DMC electrolyte are 288, 202, 174, 156 and 104 mAh/g at current densities of 0.1, 0.2, 0.4, 0.8 and 1.6 C, respectively. When the current density is restored to 0.1 C, the discharge specific capacity still remains a high reversible capacity of 280 mAh/g, demonstrating the remarkable rate performance of the samples. The corresponding GCD curves are presented in Figure 4d. The cycling performance at 0.4 C of VS_4_ nanorods with different electrolytes are shown in Figure 4e. For the EC/DMC electrolyte, the initial discharge specific capacity of VS_4_ is 440 mAh/g, corresponding to an initial coulombic efficiency (CE) of 70%. The irreversible capacity loss may be caused by the formation of an SEI film and the pre-activation process of electrode materials. The CE of VS_4_ nanorods is close to 98% in the following cycles. The capacity of VS_4_ nanorods is stable (182 mAh/g) after 100 cycles, indicating outstanding cycling stability compared to other electrolytes. To further explore the electrochemical performance and charge-transfer kinetics of the three electrolytes, electrochemical impedance spectroscopy analysis (EIS) was tested by the Nyquist plots (Figure 4f). The semicircle mainly reflects the charge transfer resistance (R_c_) of the electrode, including an ohmic resistance (R_1_) and a charge-transfer process (R_c_/C_2_) of the electrolytes, as indicated in the proposed equivalent circuit. The ohmic resistance R_1_ is related to the ionic conductivity of the discharge of the materials, and the Warburg impedance (W) responds to the diffusion of Na^+^ ions [34,35]. As shown in Figure 4f, the significant difference between the three impedance spectra is that the EC/DMC (red line) has much lower (R_c_) than the EC/DEC and TEGDME. Meanwhile, it was found that the EC/DMC electrolytes had higher frequencies in the onset between the semicircle and the slopping straight line than the control cell. This is another indication for the higher reaction kinetics of the EC/DMC electrolytes. Based on these results, the improved performance by EC/DMC electrolytes can be attributed to a smaller resistance (R_c_), or in other words to an increase in the cell reaction kinetics of the EC/DMC electrolytes. All of the results demonstrate that the EC/DMC electrolyte contributes to the boosting of the Na-ion storage performance of VS_4_ nanorods. To better understand this issue, the viscosity of binary solvent EC-based electrolytes, which are proportional to the viscosity of the co-solvent (EC:DEC > EC:DMC), is discussed, while in single solvent-based electrolytes (TEGDME), the viscosity of the electrolyte is higher, which also proves that the presence of EC can allow good dissociation of the salt. In addition, the electrochemical stability of the binary solvent-based electrolyte EC/DMC is greater than that of EC/DEC. Therefore, EC/DMC is the optimal electrolyte. Because EC is a co-solvent that increases ionic conductivity by improving salt dissociation (if the dielectric constant is high) and/or by reducing the viscosity of the resulting electrolyte and thus increasing ionic mobility, its content should not be too large or too small. It should be optimal when its volume ratio to another solvent is 1:1.

**Figure 5:**
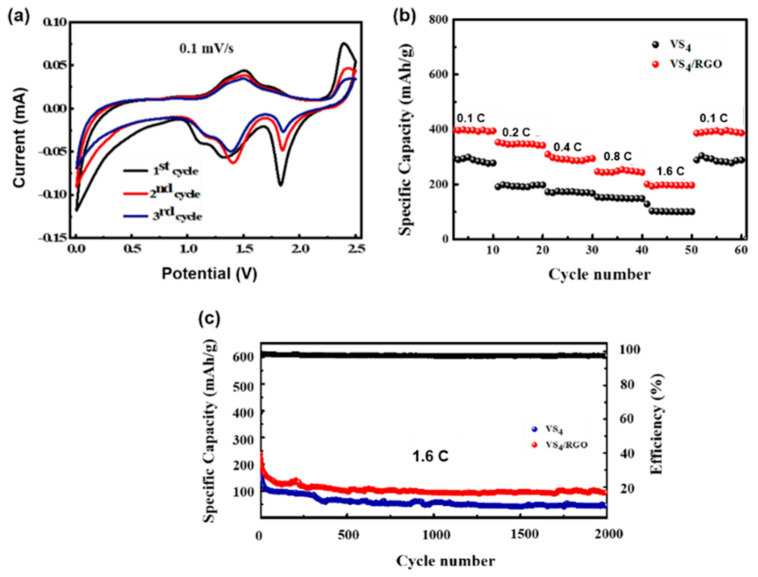
Electrochemical measurements of VS_4_/RGO nanocomposites in comparison with nanorods and RGO: (**a**) CV curves for the initial cycles at the scanning rate of 0.1 mV/s of VS_4_/RGO nanocomposites; (**b**) rate performance at the different densities; (**c**) long-term stability performance at the current density of 1.6 C.

Figure 5a presents the cyclic-voltammetry (CV) curves of the first three cycles of VS_4_/RGO as SIBs anode material, which are measured in the voltage of 0.1–2.5 V at the scan rate of 0.1 mV/s. During the initial cathodic sweep, a prominent cathodic peak is observed at 1.3 V and disappears in the subsequent cycles, which is ascribed to the formation process of the irreversible SEI film. The redox peaks in the subsequent cycles represent the multiple conversion reaction between VS_4_ and Na_x_VS_4_ [23], and the corresponding reaction equations are as follows:

VS_4_ + x Na^+^+x e^−^ → Na_x_VS_4_ (1)

Na_x_VS_4_ + (8 − x) Na^+^ + (8 − x) e^−^ → 4 Na_2_S + V (2)

The CV profiles of VS_4_/RGO electrode materials overlap after the first cycle, indicating high chemical reversibility. The galvanostatic charge/discharge profiles of pure VS_4_ nanorods, VS_4_/RGO nanocomposites and RGO in the voltage range of 0.1–2.5 V at a current density of 0.1 C are shown in Figure S2 (Supplementary Material). The VS_4_/RGO electrode exhibits superior sodium storage capacity (395 mAh/g) compared to VS_4_ nanorods (300 mAh/g) and RGO (35 mAh/g) anodes. There are two advantages for the outstanding Na-ion storage performance of VS_4_/RGO nanocomposites: RGO, as one of the most desirable carbon materials, can enhance the electronic conductivity of VS_4_. Moreover, the large scale RGO nanosheets with abundant oxygen-containing functional groups can provide sufficient redox active sites to effectively alleviate the aggregation of VS_4_ nanorods. When the current density is 0.4 C, for the EC/DMC electrolyte, the first-cycle discharge capacity of the VS_4_ electrode material is 571 mAh/g, which is due to the irreversible capacity loss caused by the formation of the SEI film during the first charge-discharge process (Figure S3, Supplementary Material).

The rate capabilities of VS_4_ nanorods and VS_4_/RGO nanocomposites are measured and compared at various current densities (Figure 5b). As the current density increases from 0.1 C to 1.6 C, the discharge capacity of VS_4_/RGO nanocomposites slowly delay. When returning to the initial density of 0.1 C, the average capacity of hybrid electrode materials remains about 400 mAh/g. As a consequence, Figure 5c demonstrates the cycling performance of VS_4_ and VS_4_/RGO at the current density of 1.6 C. A long-term and stable cycling performance of VS_4_/RGO nanocomposites is achieved with the discharge capacity retention of 100 mAh/g over 2000 cycles. This is closely related to the layer structure of VS_4_ nanorods dispersed uniformly on the surface of RGO nanosheets with high conductivity, which provides sufficient conductive channels and electroactive sites for fast electron transport and sodium ions insertion/extraction and increases the effective contact between the surface of the electrode material and the electrolyte, thus facilitating the redox reactions. Furthermore, the introduction of layered RGO can offer a stable substrate for superior long-term cycling performance.

**Figure 6:**
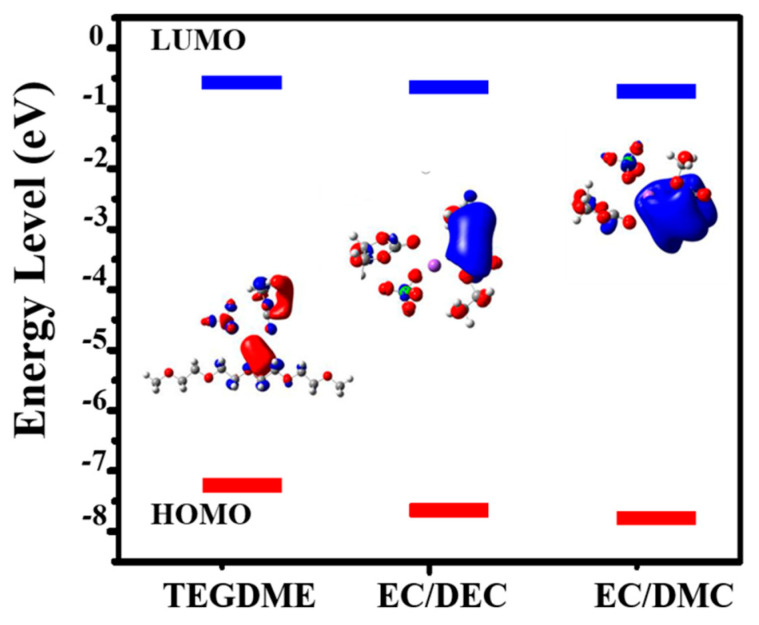
The highest occupied molecular orbital (HOMO)-lowest unoccupied molecular orbital (LUMO) energy levels of solvated Na^+^ and salt-solvent complexes in three electrolytes obtained by the Density functional theory (DFT) (inset: optimized geometrical structures).

Density functional theory (DFT) calculations were carried out to further illustrate the influence of electrolytes on VS_4_ nanorods. As shown in Figure 6, the solvation energy of 1 M NaClO_4_ in EC/DMC, EC/DEC and TEGDME electrolytes are −1.52606 eV, −1.54102 eV and −1.43974 eV, respectively (Table 1). The lower solvation energy demonstrates a weak interaction between salt and solvent, favoring Na^+^ diffusion and desolvation. For carbonate-based electrolytes (EC/DMC and EC/DEC), the values of solvation energy is very close. In addition, the LUMO energy of EC/DMC electrolyte (−0.61146 eV) is lower than that of EC/DEC (−0.66178 eV) and TEGDME (−0.74174 eV), suggesting that it is easier to reduce [32]. Electrolyte ionic conductivity is one of the most crucial factors for sodium ion storage performance [36]. Therefore, the electrical conductivity and viscosity of three electrolytes are also measured (Table S2 and Figure S4, Supplementary Materials). The highest conductivity value of 1 M NaClO_4_ in EC/DMC electrolyte is presented (7.88 mS/cm) due to its lower viscosity values as compared to other electrolytes. In conclusion, the lower viscosity of an electrolyte can improve the ionic mobility. These results indicate that an optimized EC/DMC electrolyte can facilitate Na-ion diffusion/desolvation for superior cycling stability of electrode materials.

## 3. Materials and Methods

### 3.1. Synthesis of VS_4_ Nanorods and VS_4_/RGO Nanocomposite

All chemical reagents used in the experiments were of analytical grade without further purification. In a typical fabrication process, 2 mmol ammonium vanadate (NH_4_VO_3_) was first dissolved in 30 mL deionized water/ethanol (1:1) mixed solution and stirred vigorously for 1 h at room temperature to form a homogeneous suspension, followed by the addition of 10 mmol thioacetamide (CH_3_CSNH_2_). The obtained precursor suspension was then transferred to a 50 mL Teflon-lined stainless-steel autoclave and heated at 160 °C for 12 h. After naturally cooling down to room temperature, the precipitate was filtered and washed thoroughly with deionized water and absolute ethanol for several times, and dried in a vacuum oven at 60 °C overnight. For comparison, 50 mg of graphene oxide (GO) power prepared by the modified Hummers method [37] was dispersed into the above suspension of VS_4_ nanorods and then underwent a similar procedure of solvothermal treatment. Finally, the precursors were annealed at 400 °C for 1 h to thermally reduce GO to RGO to obtain the VS_4_/RGO nanocomposite.

### 3.2. Material Characterizations

The phase purity and crystal structure of the products were investigated by X-ray diffraction on a MiniFlex 600 (Rigaku, Tokyo, Japan) with Cu Kα radiation (λ = 0.15406 nm) under the accelerated voltage and current of 40 kV and 15 mA, respectively. The morphology and microstructure of as-prepared samples were characterized by field emission scanning electron microscopy (FESEM, SU-8020) (Hitachi High-Technologies Corporation, Japan.) and transmission electron microscopy (TEM, JEM-2800) (JEOL, Tokyo, Japan) with an energy dispersive X-ray detector (EDX). Raman spectra were examined using a Renishaw in Via Raman microscope (Renishaw, Wotton-under-Edge, UK) with an excitation wavelength of 532 nm.

### 3.3. Electrochemical Measurements

Electrochemical tests were evaluated by assembling CR2032 coin type half-cells in an argon-filled glove box (O_2_ < 0.1 ppm, H_2_O < 0.1 ppm). The working electrodes were fabricated by mixing 70 wt% of as-prepared active materials, 20 wt% acetylene black and 10 wt% polyvinylidene fluoride (PVDF) binder using N-methyl pyrrolidone as solvent and the mass loading of an electrode of 0.62 mg/cm^2^. Sodium disks were cut into a round shape with 13 mm and employed as the counter electrode and glass fiber was used as the separator. The obtained homogeneous slurry was coated onto the current collector (Al foil) and dried in a vacuum oven at 60 °C overnight. In the cells, 1 M NaClO_4_ in ethylene carbonate/diethyl carbonate (EC/DEC, 1:1 *v*/*v*), ethylene carbonate/dimethyl carbonate (EC/DMC, 1:1 *v*/*v*) with the addition of 5% fluoroethylene carbonate, and tetraethylene glycol dimethyl ether (TEGDME) were used as the electrolytes for SIBs. Galvanostatic discharge/charge tests were collected on a Neware multichannel battery system between 0.1 V and 2.5 V. Cyclic voltammetry (CV) profiles were carried out using a CHI660E electrochemical analyzer (CH Instruments Inc., Shanghai, China) and electrochemical impedance spectroscopy (EIS) was performed on an Autolab electrochemical workstation.

## 4. Conclusions

In summary, VS_4_ nanorods with an average diameter of 30 nm were successfully prepared by a fast and simple one-pot solvothermal process. To further increase the electric conductivity of VS_4_ materials and improve the electron transportation, small VS_4_ nanorods were uniformly anchored into the conductive network of large-sized RGO nanosheets to obtain VS_4_/RGO nanocomposites. When tested as anode materials at the half-cell of SIBs, VS_4_ nanorods in the EC/DMC electrolyte exhibit outstanding electrochemical performance in terms of a high reversible capacity (185 mAh/g at a current density of 0.8 C), good rate capability and long cycle life (182 mAh/g after 100 cycles at 0.4 C) compared to the EC/DEC and TEGDME electrolytes. This is ascribed to the high ionic conductivity, lower solvation energy and LUMO energy for the EC/DMC electrolyte, which is favorable for Na^+^ diffusion and desolvation. Moreover, VS_4_/RGO nanocomposites can deliver a discharge capacity of 100 mAh/g after 2000 cycles at a large current density of 1.6 C, which indicates excellent long-term cycling properties compared to pure VS_4_ nanorods. The outstanding electrochemical performance of VS_4_/RGO nanocomposites is mainly attributed to the synergistic effect between the introduction of graphene, the rational synthesis of VS_4_ nanorods and the optical electrolytes, which can effectively shorten the transportation path for electrons/ions, meanwhile greatly improving the electronic conductivity and structure stability of electrode materials.

## Figures and Tables

**Table 1:** Solvation energy, HOMO and LUMO of three electrolytes.

Electrolytes	Solvation Energy (eV)	HOMO (eV)	LUMO (eV)
EC/DMC	−1.52606	−7.64646	−7.64646
EC/DEC	−1.54102	−7.53603	−0.66178
TEGDME	−1.43974	−7.12885	−0.61146

## Data Availability

Not applicable.

## Supplementary Materials

The following supporting information can be downloaded at: https://www.mdpi.com/article/10.3390/molecules27196303/s1, Figure S1: Raman spectrum of VS_4_/RGO nanocomposites; Figure S2: Galvanostatic charge and discharge profiles at the current density of 0.1 C of VS_4_/RGO nanocomposites in comparison with VS_4_ nanorods and RGO; Figure S3. Electrochemical measurements of VS_4_ nanorods with different electrolytes: the charge-discharge curves of the first cycle at the current density of 0.4 C; Figure S4. Conductivity and viscosity distribution of VS_4_ nanorods with various electrolytes; Table S1. Coulombic efficiencies of VS_4_ electrode materials under three electrolyte conditions at a current density of 0.8 C; Table S2: The electrical conductivity and viscosity of three electrolytes.

## Sample Availability

Samples of the compounds are not available from the authors.
