# Supplementary material for: Synergy Effect of High-Stability of VS4 Nanorods for Sodium Ion Battery
Source: Molecules. 2022 Sep 24;27(19):6303. doi: 10.3390/molecules27196303 (PMC9571770; doi:10.3390/molecules27196303)
Supplement: Supplementary file 1 [file molecules-27-06303-s001.zip › molecules-1902193-supplementary.pdf]

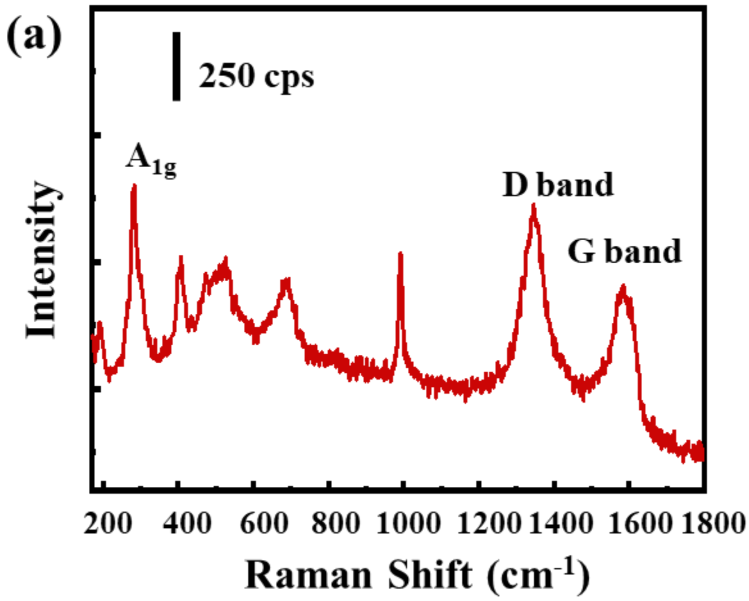

Figure S1. Raman spectrum of VS_4/RGO nanocomposites.

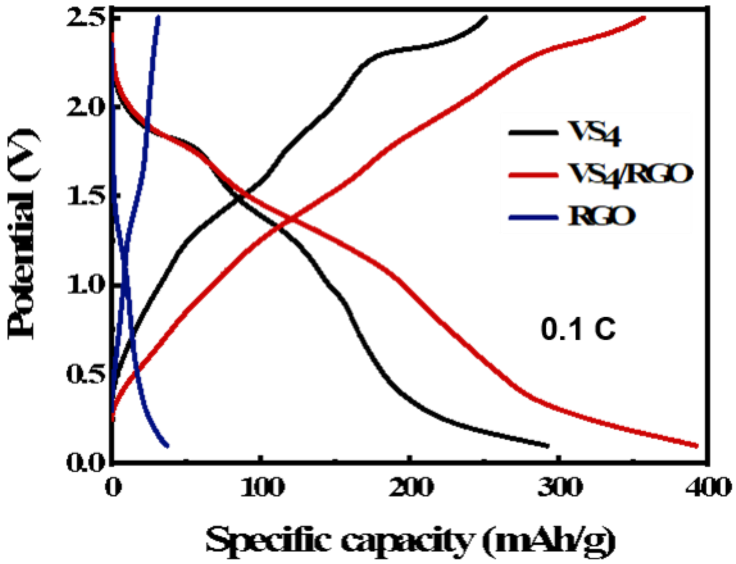

Figure S2. Galvanostatic charge and discharge profiles at the current density of 0.1 C of VS_4/RGO nanocomposites in comparison with VS_4 nanorods and RGO.

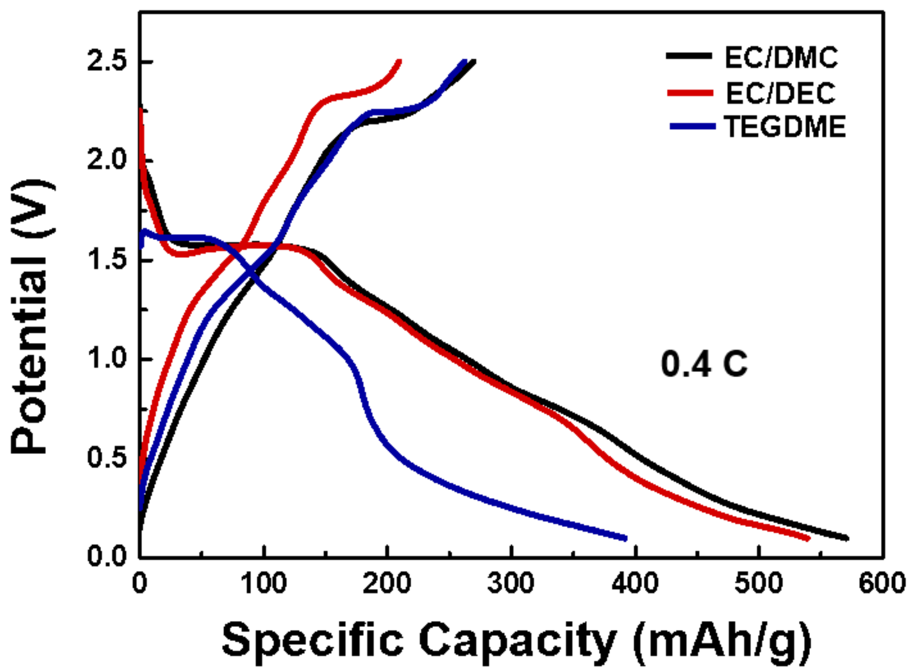

Figure S3. Electrochemical measurements of VS_4 nanorods with different electrolytes: the charge-discharge curves of the first cycle at the current density of 0.4 C.

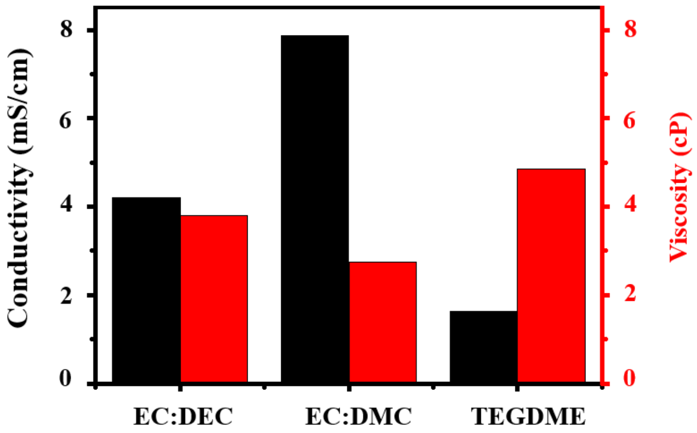

Figure S4. Conductivity and viscosity distribution of VS_4 nanorods with various electrolytes.

Table S1. Coulombic efficiencies of VS_4 electrode materials under three electrolyte conditions at a current density of 0.8 C.

**Electrolytes**	EC/DMC	EC/DEC	TEGDME
**Efficiency (%)**	98	92	90

Table S2. The electrical conductivity and viscosity of three electrolytes.

Electrolytes	Conductivity（mS/cm）	Viscosity（cP）
EC/DMC	7.88	2.75
EC/DEC	4.21	3.8
TEGDME	1.63	4.85
